# Patterns, Outcomes and Predictors of Pediatric Medical Admissions at Gadarif Hospital in Eastern Sudan

**DOI:** 10.3389/fped.2022.764028

**Published:** 2022-01-27

**Authors:** Mohammed Ahmed A. Ahmed, Imad R. Musa, Hyder M. Mahgoub, Abdullah Al-Nafeesah, Osama Al-Wutayd, Ishag Adam

**Affiliations:** ^1^Faculty of Medicine, Gadarif University, Gadarif, Sudan; ^2^Department of Medicine, Royal Commission Hospital in Al Jubail Industrial City, Al Jubail, Saudi Arabia; ^3^Department of Pediatrics, Unaizah College of Medicine and Medical Sciences, Qassim University, Unaizah, Saudi Arabia; ^4^Department of Family and Community Medicine, Unaizah College of Medicine and Medical Sciences, Qassim University, Unaizah, Saudi Arabia; ^5^Department of Obstetrics and Gynecology, Unaizah College of Medicine and Medical Sciences, Qassim University, Unaizah, Saudi Arabia

**Keywords:** morbidity, mortality, under 5 years, predictors, children

## Abstract

**Background:**

The reduction of childhood mortality is a reliable indicator of a national health system's progress and improvement. Sudan's population is still suffering from communicable diseases, with a considerably higher mortality rate among children. Efforts are therefore needed to reduce mortality and achieve the Millennium Development Goals and Sustainable Development Goals. This study was conducted to determine the morbidity, mortality and outcomes of children admitted to Gadarif Hospital in eastern Sudan.

**Method:**

A retrospective study was conducted by reviewing the medical files of pediatric patients who were admitted to Gadarif Hospital between March 1, 2019 and March 31, 2020.

**Result:**

A total of 740 medical files were reviewed. Most, 453 (61.2%) of the admissions were males. The median (interquartile range) age was 3.0 (8.0) years and 433 (58.8%) of the admissions were under 5 years of age. The median (interquartile range) of the length of hospital stay was 9.0 (12.0) days. Visceral leishmaniasis, malnutrition, severe malaria, sickle cell disease, acute watery diarrhea, severe anemia (regardless of its cause), septicemia and acute respiratory infection were the most common causes of admission. The mortality rate was 5.7%, and it was significantly higher in females than males [24/287 (8.4%) vs. 18/453 (4.0%), *P* = 0.01] and in children under 5 years [36/433 (8.3%) vs. 6/307 (2.0%), *P* < 0.001]. Malnutrition, visceral leishmaniasis, septicemia and meningitis/encephalitis were the main diseases causing death in the study population. The case fatality rate was not significantly different in malnutrition than in visceral leishmaniasis [9/93 (9.7%) vs. 7/178 (3.9%), *P* = 0.05].

**Conclusion:**

The main causes of morbidity and mortality for children admitted to Gadarif Pediatric Hospital were communicable diseases. The mortality rate was 5.7%. Females and children under 5 years were the most vulnerable groups for fatality.

## Introduction

Childhood mortality is an essential indicator to monitor child health. Mortality indicators are still considered a starting point for health status evaluation even after marked declines in mortality rates (1). Child morbidity and hospital admission are considered financial burdens to governments, health systems, and parents (2). In Africa, infections and communicable diseases are the leading causes of childhood morbidity and mortality (3). Despite the shift in the causes of mortality toward non-communicable diseases, communicable diseases remain the major causes of mortality and morbidity in middle- and low-income countries (4). Unlike the developed countries, where mortality from non-communicable diseases is escalating (5), in Sub-Saharan Africa, childhood mortality is mainly due to preventable communicable diseases (6, 7). Moreover, pediatric sepsis is a leading cause of hospital admission, with an increased risk of fatality in the African countries, including Sudan (8–10). Therefore, more research is needed to address mortality in children aged 5–9 years and in young adolescents. The World Health Organization (WHO) has reported that preventable diseases, such as respiratory tract infections, diarrheal diseases, and meningitis are responsible for about one million deaths in older children and young adolescents (11). Hence, further efforts are required to evaluate morbidities and mortalities in these age groups in order to improve outcomes. Previous studies have reported a significant variation in the leading causes of mortality between regions, gender, and age groups (4, 12). Most countries have very little information on mortality and general health conditions and, hence, it is of paramount importance to study the mortality conditions in these poor countries. There is a high rate (58.4 deaths per 1,000 live births) of childhood mortality in Sudan (13), which is one of the “least developed” countries in Africa. This study aimed to determine the patterns of morbidity and mortality and their predictors among children admitted to Gadarif Hospital in eastern Sudan.

## Materials and Methods

Medical files (paper-based) of the children admitted to Gadarif Pediatric Hospital between March 1, 2019 and March 31, 2020 were retrospectively reviewed. The hospital is a tertiary care facility that serves as a referral center in Gadarif State and is staffed with eight consultants, 10 specialists, and 25 medical doctors (registrars and residents).

### Inclusion and Exclusion Criteria

All children (aged between 1 month and 18 years) admitted to the hospital during the study period and with complete medical records were included. We excluded patients with missing information on diagnosis, age, or gender. If the patient was admitted more than once during the period of the study, the last admission was considered.

We followed the systematic random sampling technique to select the medical files to review. According to the hospital records, there were 2,273 medical files during the study period. The sampling interval (≈ 3) was assumed in dividing the all-medical files (2,273) by the calculated sample size (2,273/740 ≈ 3). Thus, the medical files were reviewed every three intervals to arrive at the required sample size (740). The subsequent medical file was taken if the selected file had incomplete data.

A seven-part questionnaire was used to collect the data, as follows: (1) socio-demographic information about the child and their family, their vaccine status, and their ward admission, (2) clinical diagnosis, (3) symptoms and signs, (4) relative investigations, (5) treatment, (6) outcome, and (7) cause of death.

### Sample Size

A sample size of 740 medical files of children was calculated based on the reported rate of death (5.6%) in a previous study in Nigeria of children admitted to hospital (3). We assumed that 55 vs. 35% and 85 vs. 65% were the rates of the females and under-fives in children who died and children who survived, respectively. The gender and the rate of under-five children were chosen because the possible difference in these variables might serve as guidance for future interventions. This sample size had an 80% power, with a precision of 5%, assuming that 10% of the files would have incomplete data.

### Statistics

The data were analyzed with the SPSS (Statistical Package for the Social Sciences) software, version 22.0. Frequency tables and percentages were generated for all the major variables of interest. The categorical variables were presented as percentages in tables, while comparisons between the variables were done using the Chi Square test. A *p*-value of <0.05 was considered statistically significant.

## Results

A total of 740 children were admitted during the study period. Of these, 287 (38.8%) were female and 453 (61.2%) were male, with a male: female ratio of 1.57:1.

The median (interquartile range) age was 3.0 (8.0) years, and 433 (58.8) were aged <5 years. The median (interquartile range) of the length of hospital stay was 9.0 (12.0) days.

**Figure 1:**
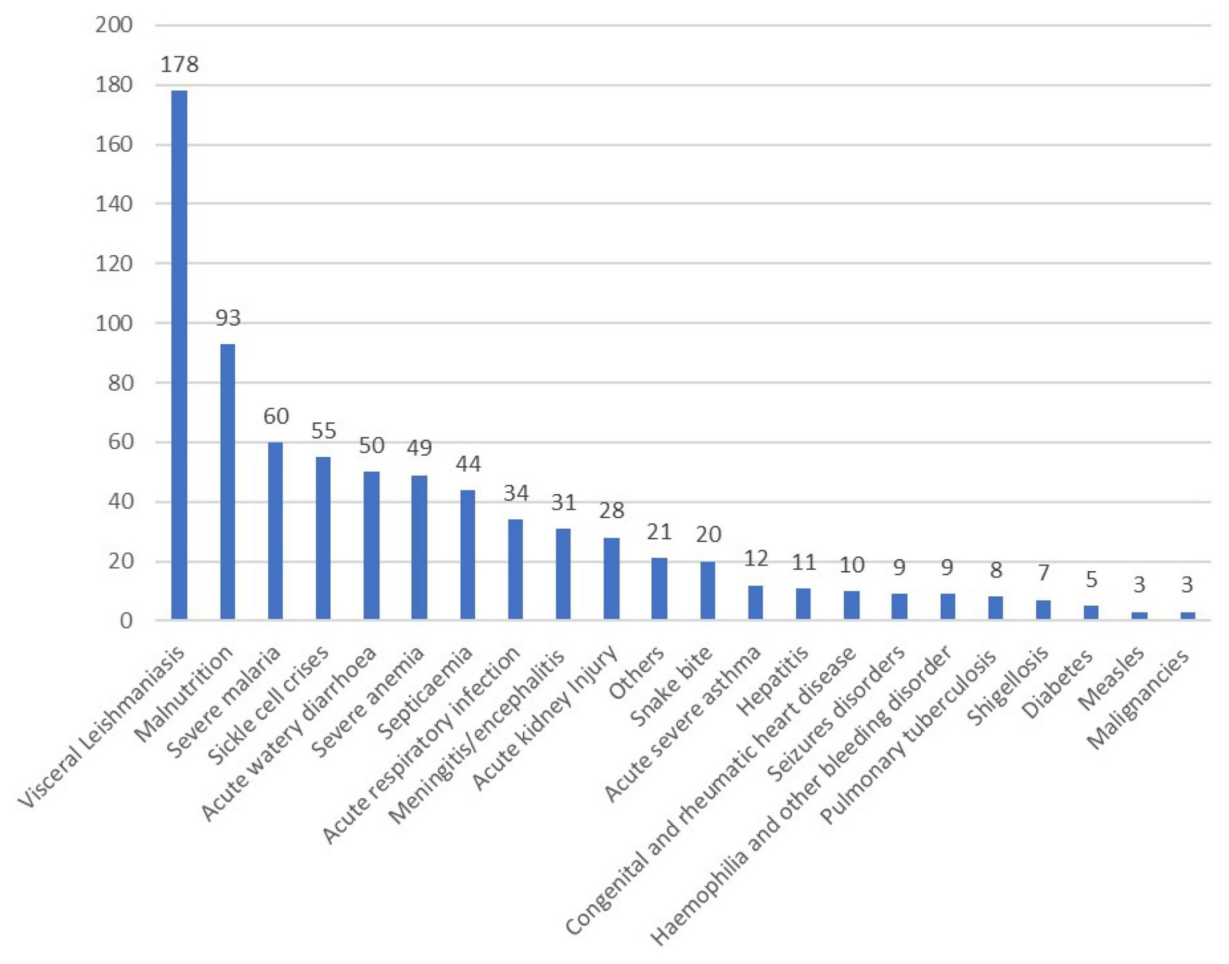
Diagnosis on pediatrics admission in Gadarif Hospital in eastern Sudan in 2020 (number = 740).

Visceral leishmaniasis, acute severe malnutrition, severe malaria, sickle cell disease, acute watery diarrhea, severe anemia (regardless to its cause), septicaemia and acute respiratory infection were the most common causes of admission (Figure 1).

Forty-two patients died, resulting in a mortality rate of 5.7%. Forty-three children (5.8%) were discharged against medical advice, 638 (86.2%) were deemed well and discharged and 17 (2.3%) were referred elsewhere. The death rate was significantly higher in females than males [24/287 (8.4%) vs. 18/453 (4.0%), *P* = 0.01] and in children under five compared to children over five [36/433 (8.3%) vs. 6/307 (2.0%), *P* < 0.001]. Acute severe malnutrition or its complications, visceral leishmaniasis, septicemia and meningitis/encephalitis were the major diseases causing death in the study population.

The case fatality rate was not significantly different for malnutrition than for visceral leishmaniasis [9/93 (9.7%) vs. 7/178 (3.9%), *P* = 0.05].

## Discussion

The main findings of the current study are that around three-fifths (61.2%) of the admissions were males, visceral leishmaniasis was the leading cause of admissions, and 5.7% of these children died. A similar finding of a higher proportion of male admissions than female admissions has been reported in neighboring Ethiopia (14). This may be explained by the underlying social factors affecting the care-seeking behaviors of family members (15). Moreover, some factors related to pediatrics admission, such as breastfeeding, have been reported to be higher in female infants (20% more likely) than in males (16).

In the current study, visceral leishmaniasis, malnutrition, severe malaria and sickle cell disease were the most common causes of admission. Previous studies have shown that severe anemia, sickle cell disease, malaria and visceral leishmaniasis were the main causes of pediatric morbidity and mortality in eastern Sudan (17, 18) and in other African countries, e.g., Nigeria, Tanzania, and Uganda (3, 19, 20).

The current study documented a mortality rate of 5.7%, similar to what has been observed previously in Sudan (5.8%) (13), as well as in Nigeria (5.7%) (3) and in Liberia (5.4%) (21). The mortality rate of children in the current study was lower than that reported in Ghana (7.12%) (22) but higher than in other African countries, e.g., Ethiopia (0.042%) (14), Malawi (3.3%.) (23), and Nigeria (4.9%) (24). The lower mortality rate in these countries may reflect a marked improvement in pediatric health facilities (14, 23). The mortality rate (8.3%) in children under five in this study was higher than the mortality (7.0%) of children under 5 years of age in Sudan (25). In contrast to this finding, a higher mortality rate (12.5%) was documented in Ethiopia in children under 5 years of age (26). It is worth mentioning that the results of our study should be cautiously compared with the results of the later ones because of the difference in socioeconomic status and other factors.

In the current study, despite the higher rate of admission for males, the death rate was significantly higher in females than in males and in children under five compared to over five, (8.4%) vs. (4.0%) and (8.3%) vs. (2.0%), respectively. This is in line with the results obtained in Tanzania (19) and in Ethiopia (15). Conversely, other studies have reported significantly higher mortality among males than females (3, 27). Although this discrepancy in gender mortality is fully explained, some have proposed a difference in immune response that is influenced by sex hormones (28). In addition, it has been previously reported that the tradition in Africa is to take care of male children first before female children (29).

In this study, malnutrition was the leading cause of mortality, with a case fatality rate of 9.7%, which was lower than the case fatality rate (12.5%) of malnutrition in Omdurman (part of the capital of Sudan) (30). However, a lower rate (3.7%) of case fatality of malnutrition has been reported in central Sudan (31) and in eastern Sudan (32). Visceral leishmaniasis was the second leading cause of morbidity and mortality in this study. Gadarif is an endemic area for this particular disease, which represents a high pediatric health burden (17, 18). Visceral leishmaniasis is also endemic in some areas of Sudan in general (33) and is frequently associated with severe anemia requiring blood transfusion (17). Severe anemia, another major cause of mortality in this study, can result from secondary causes, such as sickle cell disease, malaria, visceral leishmaniasis, severe acute malnutrition, snake bites, and sepsis (17). Severe-to- moderate anemia was also reported to be the main cause of admission to hospital among Ghanaian children (34).

Our study documented a considerably higher morbidity and mortality rate related to malaria infection. Malaria is an endemic disease that is associated with a significant mortality rate (5.3%) (35). Children are the most vulnerable age group for this disease, with a high mortality rate in Burundi (36) and Kenya (37, 38).

Diarrhea was a further cause of morbidity and mortality in this study. It is considered a major childhood medical problem in other areas in Sudan (39, 40) and in some African countries (41). Despite tremendous efforts, through a global initiative, to modify diarrheal-related fatalities in the last two decades, it is still one of the top two deadly diseases (72%) in children under the age of two in Sub-Saharan Africa in particular (42, 43). The common causes for diarrheal infection in this vulnerable group are enteric pathogenic rotavirus (44) and bacterial infections (40, 45).

In Sudan, the mortality rate may be explained by the poor distribution of health facilities in rural areas, family poverty, and the lack of hygienic and safe environments for children (46). Other reasons include low socioeconomic status and low educational levels of mothers (47, 48). Violence and war are additional risk factors that increase mortality in this vulnerable group of children (48). Similar risk factors affecting mortality have been demonstrated in several African countries, such as the preceding birth interval, family size, birth type, breastfeeding status, source of drinking water, maternal and child health services, mother's educational level, herbal medication use, sex of the child, and socioeconomic status (42, 49, 50). On the other hand, improvements in socio-demographic status, maternal health, governance, and financial status are likely to be associated with a reduction in the mortality rate (50). Furthermore, there is a growing body of evidence demonstrating that a sufficient density of distributed healthcare workers and health services can have a rapid and positive impact on neonatal and young child mortality and ultimately improve the child survival rate dramatically (51).

It is worth mentioning that human immunodeficiency virus (HIV) was reported as the top cause of death in other African countries (5), but a low incidence rate (0.4%) of HIV was reported in the hospital under study (52).

The limitations of this study are its cross-sectional design and that the data were collected only from major regional hospitals. The use of the incidence rate, a more precise measure, would be ideal to estimate the true at-risk population in etiological research. The study had some challenges, including incomplete reporting, as the hospital was unable to report cases in some years due to the lack of a computer-based system. Due to the retrospective nature of the study, some important factors, such as intubation or the use of respiratory support, were missing. In addition, some patients might have been seen in other health facilities or discharged against medical advice, and death may have occurred within the first month afterdischarge.

## Conclusion

Sudan, similar to other developing countries where communicable diseases are the most prevalent causes for morbidity and mortality among children, has a high pediatric mortality rate. Hence, many more efforts are required to achieve the Millennium Development Goals and Sustainable Development Goals in this region.

## Data Availability Statement

The raw data supporting the conclusions of this article will be made available by the authors, without unduereservation.

## Ethics Statement

The study received ethical approval from the Research Board at the Faculty of Medicine, University of Gadarif, Sudan (the reference number is 2018/11).

## Author Contributions

MA and IM made a significant contribution to the data collection and data analysis. OA-W and IA conceived the study and made substantial contributions to the study design and data interpretation. HM contributed to the data collection and drafting of the manuscript. AA-N conceived the study and played a major rule in the study design, data collection, and drafting of the manuscript. All authors read and approved the final version of the manuscript.

## Conflict of Interest

The authors declare that the research was conducted in the absence of any commercial or financial relationships that could be construed as a potential conflict of interest.

## Publisher's Note

All claims expressed in this article are solely those of the authors and do not necessarily represent those of their affiliated organizations, or those of the publisher, the editors and the reviewers. Any product that may be evaluated in this article, or claim that may be made by its manufacturer, is not guaranteed or endorsed by the publisher.

## Abbreviations

WHO: The World Health Organization
SPSS: Statistical Package for the Social Sciences
SD: Standard Deviation
HIV: human immunodeficiency virus.
